# The Interaction Between Fatty Acid Desaturase-2 (FADS2) rs174583 Genetic Variant and Dietary Quality Indices (DASH and MDS) Constructs Different Metabolic Phenotypes Among Obese Individuals

**DOI:** 10.3389/fnut.2021.669207

**Published:** 2021-06-07

**Authors:** Mahdieh Khodarahmi, Leila Nikniaz, Mahdieh Abbasalizad Farhangi

**Affiliations:** ^1^Department of Community Nutrition, Faculty of Nutrition and Food Science, Tabriz University of Medical Sciences, Tabriz, Iran; ^2^Tabriz Health Services Management Research Center, Tabriz University of Medical Sciences, Tabriz, Iran

**Keywords:** diet quality, obesity, gene-diet interaction, fatty acid desaturase (FADS) gene, cardio-metabolic risk

## Abstract

**Background and Aim:** Genetic variation in fatty acid desaturases (FADS) has previously been linked to several diet-related diseases. We aimed to determine whether the FADS2 rs174583 variant interacts with the Dietary Approach to Stop Hypertension (DASH) score and Mediterranean dietary score (MDS) to influence cardio-metabolic risk factors among obese adults.

**Methods:** This cross-sectional study was performed among 347 apparently healthy obese adults (aged 20–50 years). Dietary quality indicator scores (DASH and MDS) were generated using a validated 147-item Food Frequency Questionnaire (FFQ). The FADS2 rs174583 variant was genotyped by polymerase chain reaction-restriction fragment length polymorphism (PCR-RFLP). The gene–diet interaction was analyzed by the ANCOVA multivariate interaction model.

**Results:** A significant interaction was observed between rs174583 and adherence to the DASH score in relation to serum triglyceride (TG) concentration among the female group (*P*_Interaction_ = 0.046); CT-genotype carriers who were assigned to the second tertile of DASH compared with those in the first tertile had a lower TG level (*P* < 0.05). Another significant interaction was revealed between adherence to MDS score and rs174583 polymorphism on serum glucose levels (*P*_*Interaction*_ = 0.044); the lowest mean of glucose level was observed in homozygous minor subjects (TT) in the third tertile of MDS, in comparison with other tertiles of this dietary index (*P* < 0.05). There was a similar significant interaction between DASH and rs174583 in relation to diastolic blood pressure (*P*_Interaction_ = 0.038) among the male group. Additionally, a significant positive association was found between TT genotype and odds of having high TG both in the crude (OR, 3.21; 95% CI, 1.02–10.14) and adjusted (OR, 3.58; 95% CI, 1.07–11.97) models, taking into account different confounders.

**Conclusion:** Adherence to the dietary quality indicators (DASH and MDS) modified the relationship between FADS2 rs174583 polymorphism and cardio-metabolic risk factors in obese subjects. Prospective cohort studies are needed to confirm the results of our study.

## Introduction

Obesity, as a consequence of nutrition transition, has emerged as a major global health problem and is now a worldwide epidemic (1). Based on World Health Organization (WHO) estimation in 2016, over 650 million adults were obese. Iran, like many other developing countries, is joining the global obesity epidemic; the national prevalence rate of obesity among Iranian adults was 22.7% in 2016 (2). Obesity is known to be a complex and preventable disorder which is highly associated with a high risk of chronic diseases such as metabolic syndrome, type 2 diabetes mellitus (DM2), cardiovascular disease (CVD), and certain cancers (3). Obesity is also a cause of cardio-metabolic risk factors which are related to the risk of several abovementioned health problems (4). On the other hand, epidemiological studies have demonstrated that plasma fatty acid composition plays an important role in the development of chronic diseases, such as obesity and its related consequences (5). Recently, CVD has been considered as the leading cause of death in Iran (6), and it has been estimated that 50% of all deaths per year and 79% of deaths associated with chronic diseases are attributed to CVD (7, 8). Obesity, as a multifactorial condition, is caused by genetic makeup, environmental factors, and the synergistic interaction between them (9). In other words, major modifiable environmental factors such as diet can modify the genetic predisposition to various disorders. Over the last two decades, approximately 140 obesity susceptibility genes have been found to be related to weight gain or obesity (10) and generally 40–60% of the variations in obesity-related phenotypes have been explained by hereditary factors (11). Considering the broad roles that polyunsaturated fatty acids (PUFAs) have in the development of non-communicable diseases (12), recent candidate gene studies have concentrated on the contribution of genetic variants in fatty acid desaturases (FADS) to changes in the profile of endogenous fatty acids (13). The FADS1 and FADS2 genes which are localized on chromosome 11 (11q12–q13.1) encode delta-5 (D5D) and delta-6 fatty acid desaturases (D6D), respectively (14, 15). Altered activities of D5D and D6D, key enzymes in PUFA metabolism, were described in obesity and a number of chronic diseases such as metabolic syndrome, type 2 diabetes, cardiovascular diseases, and some malignancies (16). Recently, single-nucleotide polymorphisms (SNPs) in FADS1 and FADS2 genes have been associated with altered activity of D5D and D6D enzymes and also risk of obesity (17) and its-related conditions, such as elevated level of triglyceride, decreased high-density lipoprotein cholesterol (HDL-C) concentrations (18), and a higher risk of coronary disease (19) and type 2 diabetes (20). Nevertheless, there are some inconsistencies in the outcomes of SNP association studies which may, in part, be explained by interactions with environment factors, such as dietary influences.

Several socio-demographic factors such as sex, age, and socioeconomic status (SES) (21) as well as psychological factors (22) have indicated to play a main role in the development of obesity and its related health outcomes. Among psychosocial stressors, depression and anxiety have received special attention in the past years and a growing number of studies have reported a strong and independent relationship between these psychological disorders and the development of chronic diseases such as CVD (23). It has been suggested that chronic diseases are influenced by the interaction between mental health and various unhealthy behaviors such as poorer dietary quality and physical inactivity (24). Diet, as an important lifestyle factor, plays a crucial role in the prevention and treatment of obesity and its complications (25). In recent years, a dietary pattern approach that represents a broader picture of food and nutrient intake and accounts for the complexity of the diet has gained much attention in disease prevention, compared to their single dietary components (26). Among a large number of priori-defined diet quality indices that have been proposed in the nutrition literature, the Dietary Approach to Stop Hypertension (DASH) score and Mediterranean dietary score (MDS) have been widely used (27). These diet quality indices (DASH and MDS) have been frequently associated with a reduced risk of diseases, such as diabetes, CVD, and several forms of cancer (27, 28). Although a growing body of research has confirmed the significant and consistent protection provided by adherence to these healthy dietary patterns in relation to various health outcomes (29, 30), other studies have not provided clear evidence of the beneficial effects of high adherence to these diet indices (31, 32). Moreover, there is a relative shortage of knowledge on these health promotion indices in developing countries (33–35). On the other hand, responses to dietary interventions for the prevention and treatment of obesity and its related health conditions differ among various ethnicities with different genetic structures (36), suggesting the existence of gene–diet interactions which may play a main role in these interindividual inequalities. Thus, understanding the nature of gene–diet interactions is crucial in the treatment and management of obesity and its related chronic conditions and it can even be used to develop individualized effective dietary strategies. To the best of our knowledge, the interactions between adherence to the dietary quality indices (DASH score and MDS) and rs174583 polymorphism of FADS2 gene in relation to cardio-metabolic factors have not been examined. Therefore, we examined whether rs174583 polymorphism of the FADS2 gene has an association with cardio-metabolic risk factors in an obese Iranian population and whether those influences might be modulated by dietary quality indicators (DASH score, MDS).

## Materials and Methods

### Participants

This study was carried out as a cross-sectional study among 347 apparently healthy obese adults (58.2 % male, 41.8 % female) within the age range of 20–50 years. This study was performed from November 2017 to October 2018 in Tabriz city, one of the major cities in the northwest part of Iran. Study participants were recruited using the convenience sampling method through announcements and posters placed in healthcare facilities and public places in the city of Tabriz. These announcements contained information on the inclusion criteria (good health, obesity (BMI ≥30), and ages of 20–50 years). Initially, 400 participants were willing to take part in the research. After screening for eligibility based on the inclusion and exclusion criteria, 48 individuals were excluded from the study. The exclusion criteria for the present study included pregnant, lactating, and menopausal women; medical history of chronic diseases such as cardio-vascular diseases, hypertension, hyperlipidemia, diabetes, renal diseases, hepatic disorders, and cancer; and having any recent surgery such as bariatric surgery. Additionally, people receiving any medications and supplements which had effects on weight and variables studied such as loop diuretics, corticosteroids, antidepressants, statins, and antihypertensive agents were also excluded from the study. Before the commencement of the study, the objective of the study was described for eligible participants and they had time to ask their questions to the research team. During the study, two subjects dropped out of the study because their dietary questionnaires were incomplete. We also excluded participants (*n* = 3) whose total energy intake was outside the range of 800–4,200 kcal/day as under-reports and over-reports of energy intakes (37, 38). The required sample size for the present study was calculated by considering the association between dietary quality indices and obesity as a key dependent variable. With regard to the correlation coefficient (r) of 0.25 (39), α = 0.05, and power of 80%, using G^*^Power software, the minimum sample size was calculated to be 225, and taking into account 20–25% dropout, the final sample size of 340 was considered for the study. Finally, a total of 347 available subjects who agreed to participate were enrolled in the current study and written consent was obtained from each subject before enrollment to the study. The Ethical Committee of the Tabriz University of Medical Sciences approved the study protocol (registration codes IR.TBZMED.REC.1399.874 and IR.TBZMED.REC.1396.768). Metabolic syndrome (MetS) was defined using criteria established by The National Cholesterol Education Program (NCEP) Adult Treatment Panel (ATP) III (40). Individuals were identified to have MetS if they had three or more of the following criteria: waist circumference > 102 cm (men) or 88 cm (women), blood pressure ≥ 130/85 mmHg, fasting triglyceride (TG) level ≥ 150 mg/dl, fasting high-density lipoprotein cholesterol (HDL-C) level <40 mg/dl or 50 mg/dl (women), and fasting blood sugar ≥ 100 mg/dl. The cardio-metabolic risk factors were also defined based on Adult Treatment Panel III (40).

### Dietary Assessment and Dietary Score Calculation

The habitual dietary intakes of the participants were assessed with the use of a 147-item semiquantitative food frequency questionnaire (FFQ) with prior evidence of validity and reliability (41, 42). All information was gathered by expert dietitians in a face-to-face interview with each participant. The study subjects were asked to report their frequency and amount of intake of each food item during the previous year based on daily, weekly, monthly, and yearly bases. After that, portion sizes of foods were converted to daily intakes (grams) using household measurements. Since the Iranian food composition table (FCT) (43) is incomplete, USDA FCT was also used to provide information missing from the Iranian FCT (44).

#### DASH Score

The level of conformity of the individuals' diet to the DASH diet was determined by a scale proposed by Fung et al. (45). This indicator was constructed based on eight food-group or nutrient components which were emphasized or minimized in the DASH diet. This approach strongly encourages the intake of fruits, vegetables, low-fat dairy products, nuts, legumes, and whole grains while discouraging the consumption of red and processed meats, sodium, and sweetened beverages. Sex-specific quintile intakes of the dietary components were calculated within the study population. Participants with the highest quintile of intakes of emphasized components (fruits, vegetables, whole grains, low-fat dairy, nuts, and legumes) received a score of five, while those with the lowest quintile of these intakes were given a score of one. For the remaining components in which their low intakes were desirable (sodium, red and processed meats, and sweetened beverages), a reverse-scoring method was used. Then, the scores for all components were summed up to obtain an overall DASH score (ranged 8–40). A higher total score represents a higher conformity to the DASH diet and a better nutrition quality.

#### MDS

The level of conformity of the subjects' diet to the Mediterranean dietary pattern was assessed using the MDS proposed by Trichopoulou et al. (46). The sex-specific median intakes of its components (nine items) were calculated. The scoring system was as follows: score one was given for intakes at or above the median for protective components such as vegetables, legumes, fruits and nuts, cereals, fish, and seafood, and a high ratio of monounsaturated fatty acid (MUFA) to saturated fatty acid (SFA) as well, otherwise a value of 0 was assigned. For non-protective components (like meats and dairy products), a value of 1 was assigned for intakes less than the median, otherwise a value of zero was given. As there was no reliable data on alcohol consumption in the current study, this component was omitted and the scoring system was modified with a total score of eight points. Finally, the overall score was reported as a summation of all component scores ranging between 0 (minimal adherence to the Mediterranean diet) and 8 (maximum adherence).

### Socio-Demographic, Anthropometric, and Blood Pressure Assessments

Information of socio-demographic variables such as age, gender, marital status, smoking, medical history, and socioeconomic status (SES) was obtained by a trained interviewer. To determine the SES, questions on occupational position, educational status, family size, and house ownership were considered as individual indicators. The total score determined by summing the three scale scores was categorized into three groups: low, middle, and high. The level of physical activity was estimated using a short version of the International Physical Activity Questionnaire (47). Body weight and height were directly measured using a Seca scale (Seca, Germany) and a tape measure with a precision of 100 g and 0.1 cm, respectively, while the subjects were in light clothing without shoes. Bioelectrical impedance analysis (BIA) technology (Tanita, BC-418 MA, Tokyo, Japan) was applied to assess the body composition. Waist circumference (WC) was measured using a flexible inelastic tape to the nearest 0.1 cm, at the narrowest level without applying any pressure to the body. Blood pressure (BP) measurements were conducted using a standard mercury sphygmomanometer twice after a 15-min rest in a sitting position. The average of the two measurements was reported as the participants' BP.

### Mental Health and Appetite Assessment

To assess the severity of the emotional disturbance of participants, they were asked to complete the Depression, Anxiety and Stress Scale-21 Items (DASS-21) questionnaire which has been previously validated in Iran (48, 49). For example, Sahebi et al. reported the Cronbach's alpha of the Iranian version of DASS-21 to be 0.77 for depression, 0.79 for anxiety, and 0.78 for stress (48). DASS-21 consists of three categories of seven-item self-report scales which measure the negative states of depression, anxiety, and stress. Items in this questionnaire were scored based on a Likert scale from zero (“did not apply to me at all”) to 3 (“applied to me very much or most of the time”). A total score for every scale was computed by summing up the scores for the relevant items and multiplying them by two which could range from 0 to 42. Based on cutoff scores which have been proposed by Lovibond and Lovibond, all the participants were divided into five groups: normal, mild, moderate, severe, and extremely severe depression, anxiety, and stress (50). Higher scores of each subcategory indicated a greater degree of mood disruption.

A 100-mm Visual Analog Scale (VAS) questionnaire was applied to assess appetite sensation. This questionnaire, which has been validated in literature, includes questions about feelings of hunger, satiation, fullness, prospective food consumption, thirst, and the desire to eat something sweet, salty, or fat (51). The participants completed this scale by placing a slash on the 100-mm horizontal line corresponding to their feelings, and subsequently quantification of the measurement was performed by measuring the distance from the left side of the line to the mark.

### Biochemical Assessments

Fasting venous blood samples were obtained from all participants and then were centrifuged (10 min at 4,500 rpm, 4°C) to separate serum. Aliquots were frozen at−80 C until analysis. Serum triglyceride (TG), total cholesterol (TC), glucose, and high-density lipoprotein cholesterol (HDL-C) were assayed using commercial kits (Pars Azmoon, Tehran, Iran). Serum insulin was also analyzed with commercially available enzyme-linked immunosorbent assay kits (Bioassay Technology Laboratory, Shanghai Korean Biotech, Shanghai City, China) according to the manufacturer's instructions. The concentration of low-density lipoprotein cholesterol (LDL) was calculated using the Friedewald formula (52). Atherogenic index of plasma (AIP) was determined as the base 10 logarithm of TG to HDL-C ratio (53). Homeostasis model assessment-insulin resistance index (HOMA-IR) and quantitative insulin sensitivity check index (QUICKI) as indicators of insulin resistance were calculated based on the standard formula (54, 55).

### Genotyping

**Figure 1:**
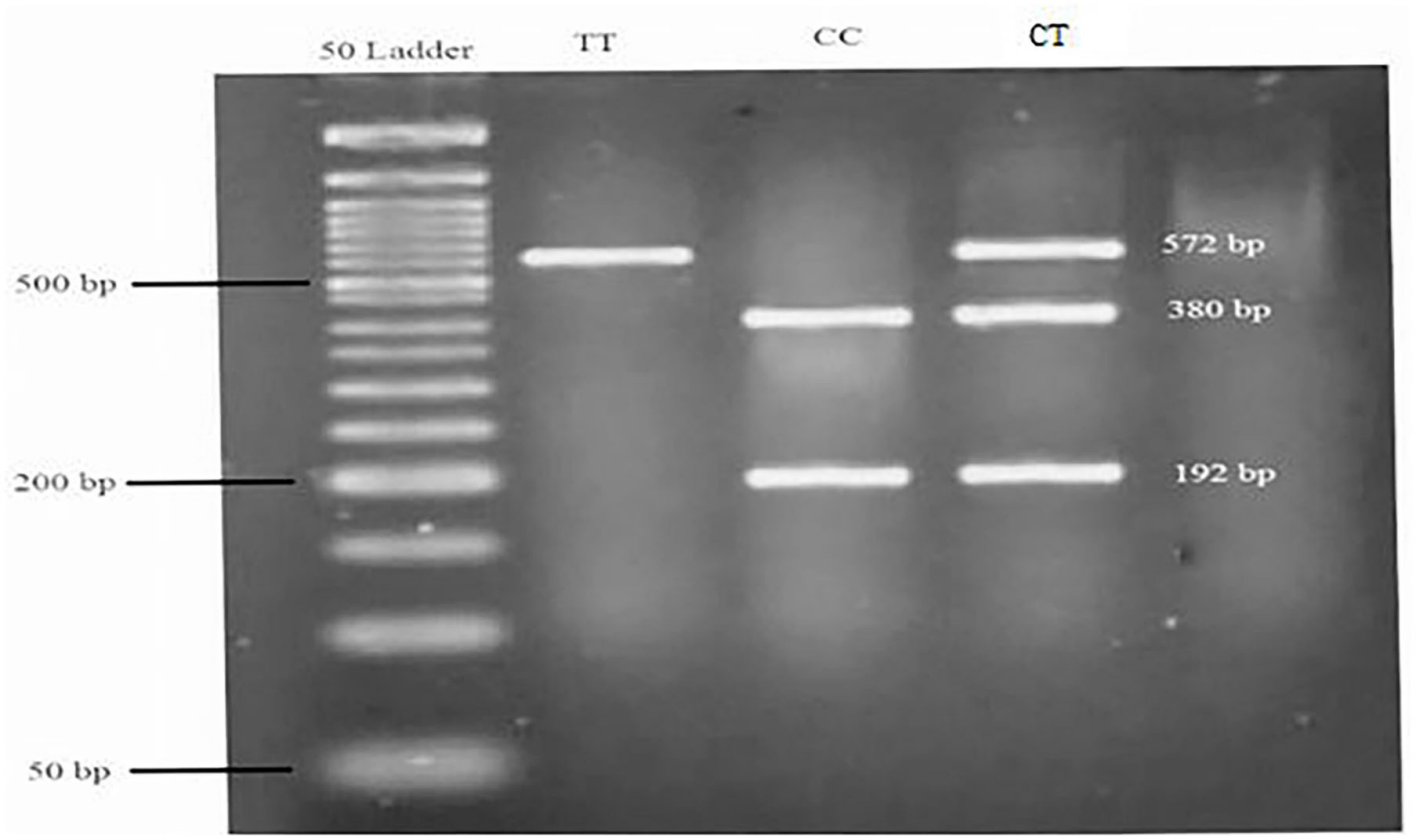
Genotyping of the FADS2 rs174583 variant by TauI PCR-RFLP analysis. A 50-bp ladder was used to determine the length of the digested products. TT = homozygous mutated (572 bp), CC = homozygous wild-type (380 and 192 bp), CT = heterozygous mutated (572, 380, and 192 bp).

Genomic DNA was extracted from whole blood using the chloroform technique, and a NanoDrop ND-1000 spectrophotometer was used to investigate the concentration and purity of extracted DNA. All available DNAs were expected to be genotyped for rs174583. SNP rs174583, which is located in the 61842278 position of chromosome 11 in the intron of FADS2, was genotyped by the polymerase chain reaction-restriction fragment length polymorphism (PCR-RFLP) method. The PCR was completed using primers with the following sequence: forward, 5′ AGGAAGCAGACCACAGAGTC 3′; reverse, 5′ TCCTTCGTCTGGTGTCTCAG 3′. PCR reactions were performed in a final volume of 10 μl which contains 5 μl Master Mix (Ampliqon, Denmark), 2 μl extracted DNA, 1 μl primers, and 2 μl distilled water. The PCR cycles in a DNA thermocycler (Bio-Rad T100 Thermal Cycler) were optimized to 95 °C for 10 min of initial denaturation; amplification consisted of 35 cycles at 95 °C for 15 s (denaturation), and annealing at 60 °C for 20 s and 50 s of extension at 72 °C and final extension occurred at 74 °C for 10 min. According to the restriction sites on the sequence of the amplified DNA, TauI (cat. num. ER1652, USA) as a restriction enzyme was used to digest amplified DNA. Afterward, all digested PCR products were visualized by green staining gel electrophoresis on 1.5% agarose gel in a Gel Doc system (U.V.P Company, Cambridge, UK). As shown in Figure 1, after electrophoresis, the T allele appeared as a 572-bp fragment whereas the C allele was cleaved by the restriction enzyme and appeared as fragments with lengths of 192 and 380 bp.

### Statistical Analysis

Sample distributions were tested for normality according to descriptive measures such as coefficients of skewness and kurtosis, mean, and standard deviation (56). The data were presented as means ± standard deviations (SD) for normally distributed continuous variables, frequencies or percentages for categorical variables, and the median (25 and 75 percentiles) for skewed continuous variables. Sex-stratified one-way analysis of variance (ANOVA) and chi-square tests were used to compare continuous and discrete variables across different tertile categories of dietary quality indices. Multinomial logistic regression in different models was utilized to estimate the associations between diet quality indices and rs174583 genotypes, as well as the association of this variant with odds of MetS and cardio-metabolic risk factors. Gene–diet interactions were tested by analyses of covariance using the General Linear Model procedures, after adjusting for confounding factors (age, physical activity, SES and WC). All of these gene–diet interaction analyses were conducted based on sex categories, and then significant interactions were depicted as plot to help their illustration. The *P*-value <0.05 was considered as statistically significant. Statistical Package for Social Sciences (SPSS, Inc., Chicago, IL, version 21) was used for all statistical analyses.

## Results

The demographic, anthropometric, clinical, biochemical, and genetic characteristics of all participants are summarized in Table 1. The mean (± SD) age of participants was 38.08 ± 7.49 years, and the mean (± SD) WC was 108.89 ± 9.90 cm. More than one half of the individuals were male (58.2 %), and 86.0 % were married. In relation to physical activity, 47.8 % of the subjects had low physical activity and more had middle SES (52.90%). It was found that 56.1 % of participants had MetS and the overall prevalence of rs174583 genotypes was 37.8, 51.9, and 10.3 for CC, CT, and TT, respectively (Table 1). Daily intakes (g/day) of macronutrients, micronutrients, and several food groups in relation to diet quality indices (MDS and DASH score) among study participants are presented in Table 2. The mean (± SD) energy intake was 3049.91 ± 1077 kcal/d. For the total sample, the mean (± SD) DASH score was 23.97 ± 4.27 points and the MDS score was 4.01± 1.43 points. The distributions of the study population regarding general characteristics and clinical, genetic, and biochemical parameters across the tertiles of dietary quality indices among men and women are presented in Tables 3, 4. Among men, in comparison with the lowest tertile, those who were assigned to the top tertile of the DASH score were older (*P* = 0.008) and were less likely to have higher severity of mental health disorders such as depression (*P* = 0.049) and stress (*P* = 0.004). The distribution of SES for men was significantly different among tertile of MDS (*P* = 0.037); those who had a higher level of SES were assigned to the second tertile of this index. Additionally, men in the third tertile of the DASH score compared with the first tertile had lower AIP (*P* = 0.032) and TG (*P* = 0.050) levels. No significant differences were observed in the frequencies of genotypes across different tertiles of diet quality indices (MDS and DASH) (*P* > 0.05). Among women, no statistical significant difference was found regarding qualitative and quantitative variables across tertile categories of dietary quality indices (Table 4). Also, we did not observe statistically significant differences in genotype frequencies of the FADS2 rs174583 polymorphism across MDS and DASH score tertiles (*P* > 0.05) in this group.

**Table 1:** Characteristics of the study participants.

	**%**	**Mean (SD) or Median (25 and 75 percentiles)**
Age (y)		38.08 (7.49)
Weight (kg)		96.10 (12.87)
FM (Kg)		33.83 (9.16)
FFM (kg)		62.29 (12.38)
WC (cm)		108.89 (9.90)
Sex		
Male	58.20	
Female	41.80	
**Physical activity level, (%)**		
Low	47.80	
Moderate	27.70	
High	24.50	
**Marital status, (%)**		
Married	86.00	
Single	14.00	
**SES**, ***n*** **(%)**		
Low	2.70	
Middle	52.90	
High	44.40	
**Stress**, ***n*** **(%)**		
Normal	40.4	
Mild	17.0	
Moderate	23.4	
Severe	14.4	
Extremely severe	4.8	
**Anxiety, (%)**		
Normal	36.2	
Mild	8.5	
Moderate	25.0	
Severe	13.8	
Extremely-severe	16.5	
**Depression, (%)**		
Normal	45.7	
Mild	12.8	
Moderate	23.5	
Severe	9.0	
Extremely severe	9.0	
Appetite		33.58 (8.96)
LDL-C, (mg/dl)		119.47 (30.91)
HDL-C, (mg/dl)		44.97 (8.85)
Cholesterol, (mg/dl)		188.43 (33.73)
TG, (mg/dl)		119.97 (58.46)
AIP		0.03 (0.24)
Glucose, (mg/dl)		91.00 (85.00, 98.00)
Insulin, U/mL		13.10 (9.10, 23.30)
HOMA-IR		3.20 (1.95, 5.28)
QUICKI		0.33 (0.03)
SBP (mmHg)		115.64(16.44)
DBP (mmHg)		76.33 (12.32)
Mets (%)	56.1	
**FADS2 (%)**		
CC	37.8	
CT	51.9	
TT	10.3	

*WC, waist circumference; SES, socio-economic status; HOMA-IR, homeostasis model assessment of insulin resistance; LDL-C, low density lipoprotein cholesterol; HDL, high-density lipoprotein-cholesterol; SBP, systolic blood pressure; DBP, diastolic blood pressure; TG, triglyceride; QUICKI, quantitative insulin sensitivity check index; AIP, athrogenic index of plasma; MetS, metabolic syndrome; FM, fat mass; FFM, fat free mass; FADS, fatty acid desaturase*.

**Table 2:** Daily dietary intake of macronutrients, energy and several food groups in relation to MDS and DASH score among study participants.

**Variables**	**Mean (SD) or Median (25 and 75 percentiles)**
**Macronutrients**	
Carbohydrate (g/day)	440.02 (164.76)
Protein (g/day)	97.86 (34.67)
Fats (g/day)	108.04 (46.85)
Energy intake (kcal/day)	3042.91 (1077.89)
Fiber (g/day)	58.03 (42.30, 92.44)
DASH score [total score (8–40)]	23.97 (4.27)
MDS [total score (0–8)]	4.01 (1.43)
Fruits (g/day)	444.03 (289.52, 751.85)
Vegetables (g/day)	320.53 (198.02, 439.48)
Nuts (g/day)	10.98 (5.01, 23.72)
Legumes (g/day)	49.13 (28.70, 73.33)
Red and processed meat (g/day)	49.05 (27.53, 78.91)
Cereals (g/day)	523.44 (355.91, 701.92)
Fish and seafood (g/day)	6.37 (1.91, 13.767)
Dairy products (g/day)	260.62 (140.26, 435.56)
MUFA/SFA	1.21 (0.31)

*MUFA, monounsaturated fatty acid; SFA, saturated fatty acid, MDS, Mediterranean diet score; DASH, dietary approach to stop hypertension*.

**Table 3:** Socio-demographic and anthropometric characteristics and cardio-metabolic risk factors according to the tertiles of dietary quality indices in men.

	**DASH**				**MDS**			
	**T1**	**T2**	**T3**	P^*^	**T1**	**T2**	**T3**	P^*^
Age (y)	36.41 (5.91)	38.11 (6.90)	41.67 (6.00)	**0.008**	37.91 (6.14)	38.50 (6.59)	38.63 (9.04)	0.969
Weight (kg)	103.60 (10.39)	102.52 (9.34)	99.65 (11.47)	0.617	102.06 (9.84)	102.53 (10.82)	101.48 (9.25)	0.960
FM (Kg)	30.35 (7.27)	28.71 (6.68)	28.42 (8.01)	0.644	28.93 (7.83)	29.43 (6.76)	29.56 (7.86)	0.952
FFM (kg)	73.26 (5.24)	73.86 (7.57)	71.24 (5.36)	0.477	73.14 (6.58)	73.15 (6.49)	71.93 (4.46)	0.961
WC (cm)	114.31 (8.05)	112.47 (6.29)	112.57 (8.30)	0.644	113.49 (8.01)	112.90 (7.29)	113.50 (6.41)	0.926
Physical activity level, (%)				0.808				0.763
Low	35.3	29.4	35.3		41.2	44.1	14.7	
Moderate	46.9	40.6	12.5		34.4	62.5	3.1	
High	26.7	50.0	23.3		30	60	10	
Marital status, (%)				0.682				0.620
Married	35.8	39.5	24.7		34.6	55.6	9.8	
Single	40.0	40.0	20.0		40.0	53.3	6.7	
**SES**, ***n*** **(%)**								
Low	0.0	0.0	0.0	0.371	0.0	0.0	0.0	**0.037**
Middle	30.0	43.3	26.7		20.0	66.7	13.3	
High	40.0	38.5	21.5		43.1	49.2	7.7	
**Stress**, ***n*** **(%)**				**0.004**				0.481
Normal	21.7	45.7	32.6		26.1	65.2	8.7	
Mild	41.2	47.1	11.8		52.9	35.3	11.8	
Moderate	47.1	29.4	23.5		47.1	47.1	5.9	
Severe	60.0	20.0	20.0		20.0	60.0	20.0	
Extremely severe	66.7	33.3	0.0		50.0	50.0	0.0	
Anxiety, (%)				0.788				0.541
Normal	35.7	42.9	21.4		33.3	57.1	9.5	
Mild	66.7	16.7	16.7		50.0	33.3	16.7	
Moderate	22.7	36.4	40.9		36.4	54.5	9.1	
Severe	35.7	42.9	21.4		14.3	71.4	14.3	
Extremely-severe	50.0	41.7	8.3		58.3	41.7	0.0	
Depression, (%)				**0.049**				0.647
Normal	27.8	40.7	31.5		31.5	59.3	9.3	
Mild	41.7	41.7	16.7		41.7	50.0	8.3	
Moderate	55.6	33.3	11.1		38.9	55.6	5.6	
Severe	33.3	50.0	16.7		33.3	50.0	16.7	
Extremely severe	50.0	33.3	16.7		50.0	33.3	16.7	
Appetite	35.09 (11.31)	34.74 (8.87)	33.96 (7.83)	0.908	33.12 (9.77)	35.43 (9.31)	36.11 (10.23)	0.490
LDL-C, (mg/dl)	122.61 (27.08)	122.69 (29.83)	113.79 (22.70)	0.201	119.14 (24.18)	122.70 (29.51)	113.95 (27.22)	0.471
HDL-C, (mg/dl)	41.88 (6.75)	44.46 (9.28)	40.62 (6.24)	0.220	41.71 (6.41)	43.24 (8.93)	42.75 (6.58)	0.675
Cholesterol, (mg/dl)	192.09 (28.44)	191.30 (35.83)	183.14 (25.87)	0.404	188.18 (27.86)	192.18 (33.83)	181.00 (26.24)	0.601
TG, (mg/dl)	134.00 (106.00, 194.00)	104.50 (76.25, 138.50)	121.00 (85.00, 168.00)	**0.050**	119.00 (86.50, 174.50)	116.00 (83.00, 164.50)	129.00 (85.50, 177.00)	0.814
AIP	0.16 (0.22)	0.02 (0.27)	0.12 (0.22)	**0.032**	0.11 (0.23)	0.07 (0.26)	0.07 (0.22)	0.753
Glucose, (mg/dl)	90.00 (84.00, 101.00)	91.00 (86.00, 99.25)	96.00 (85.00, 108.00)	0.345	91.00 (86.00, 107.00)	91.00 (85.00, 97.00)	94.00 (83.00, 101.50)	0.813
Insulin, U/mL	13.30 (9.00, 23.30)	12.00 (9.37, 20.48)	9.40 (8.40, 16.90)	0.275	12.15 (8.85, 23.63)	12.00 (9.00, 19.00)	9.40 (8.35, 21.85)	0.825
HOMA-IR	3.22 (1.94, 5.03)	2.87(2.25, 4.57)	2.04 (1.78, 4.79)	0.451	3.06 (1.98, 5.26)	2.84 (1.95, 4.64)	2.51 (1.70, 5.09)	0.771
QUICKI	0.33 (0.03)	0.32 (0.02)	0.34 (0.03)	0.401	0.33 (0.03)	0.33 (0.03)	0.34 (0.03)	0.796
SBP (mmHg)	120.00 (105.00, 130.00)	120.00 (110.00, 130.00)	110.00 (110.00–120.00)	0.480	120.00 (110.00, 130.00)	120.00 (107.50, 130.00)	110.00 (110.00, 125.00)	0.857
DBP (mmHg)	77.50 (12.57)	77.30 (10.04)	70.43 (16.61)	0.143	76.76 (11.67)	75.38 (14.34)	74.38 (8.63)	0.816
Mets (%)	41.5	31.7	26.8	0.815	43.9	48.8	7.3	0.150
FADS2 (%)				0.756				0.365
CC	37.1	28.6	34.3		28.6	54.3	17.1	
CT	41.0	48.7	10.3		48.7	46.2	5.1	
TT	30.0	30.0	40.0		10.0	90.0	0.0	

*Data are presented as mean (SD) or median (25 and 75 percentiles)*.

* *Analysis of variance for continuous variables and χ^2^ test for categorical variables. WC, waist circumference; SES, socio-economic status HOMA-IR, homeostasis model assessment of insulin resistance; LDL-C, low density lipoprotein cholesterol; HDL, high-density lipoprotein-cholesterol; SBP, systolic blood pressure; DBP, diastolic blood pressure; TG, triglyceride; QUICKI, quantitative insulin sensitivity check index; AIP, athrogenic index of plasma; MetS, metabolic syndrome; FM, fat mass; FFM, fat free mass; FADS, fatty acid desaturase*.

**Table 4:** Socio-demographic and anthropometric characteristics and cardio-metabolic risk factors according to the tertiles of dietary quality indices in women.

	**DASH**				**MDS**			
	**T1**	**T2**	**T3**	P^*^	**T1**	**T2**	**T3**	P^*^
Age (y)	36.94 (8.77)	38.51 (7.93)	38.78 (8.00)	0.669	38.23 (8.78)	37.29 (8.14)	39.33 (7.60)	0.677
Weight (kg)	87.73 (8.84)	91.77 (16.56)	89.66 (8.13)	0.418	88.57 (13.92)	89.01 (10.27)	93.62 (13.47)	0.322
FM (Kg)	37.75 (6.09)	39.93 (11.06)	38.49 (5.98)	0.633	38.71 (9.37)	37.81 (6.76)	41.15 (9.62)	0.358
FFM (kg)	50.26 (3.75)	51.62 (6.52)	51.18 (3.77)	0.466	49.58 (4.99)	51.44 (4.85)	52.47 (4.94)	0.154
WC (cm)	104.45 (9.37)	104.32 (11.03)	104.70 (10.18)	0.966	103.09 (10.03)	103.95 (9.25)	108.00 (11.90)	0.207
Physical activity level, *n* (%)				0.814				0.853
Low	33.9	41.1	25.0		33.9	42.9	23.2	
Moderate	45.0	25.0	30.0		45.0	45.0	10.0	
High	31.3	50.0	18.8		25.0	56.3	18.8	
Marital status, *n* (%)				0.413				0.827
Married	35.8	42.0	22.2		35.8	44.4	19.8	
Single	36.4	18.2	45.5		27.3	54.5	18.2	
SES, *n* (%)				0.264				0.995
Low	60.0	20.0	20.0		20.0	40.0	40.0	
Middle	34.8	43.5	21.7		36.2	47.8	15.9	
High	33.3	27.8	38.9		33.3	38.9	27.8	
Stress, *n* (%)				0.377				0.723
Normal	46.7	33.3	20.0		36.7	43.3	20.0	
Mild	40.0	33.3	26.7		40.0	46.7	13.3	
Moderate	22.2	40.7	37.0		33.3	44.4	22.2	
Severe	41.2	41.2	17.6		29.4	47.1	23.5	
Extremely severe	0.0	100.0	0.0		33.3	66.7	0.0	
Anxiety, *n* (%)				0.557				0.286
Normal	34.6	38.5	26.9		50.0	34.6	15.4	
Mild	40.0	40.0	20.0		10.0	60.0	30.0	
Moderate	44.0	44.0	12.0		40.0	44.0	16.0	
Severe	33.3	16.7	50.0		25.0	41.7	33.3	
Extremely-severe	26.3	47.4	26.3		26.3	57.9	15.8	
Depression (%)				0.699				0.959
Normal	40.6	28.1	31.3		31.3	50.0	18.8	
Mild	41.7	41.7	16.7		33.3	33.3	33.3	
Moderate	34.6	38.5	26.9		42.3	46.2	11.5	
Severe	36.4	63.6	0.0		45.5	27.3	27.3	
Extremely severe	18.2	45.5	36.4		18.2	63.6	18.2	
Appetite	34.18 (7.06)	30.97 (8.56)	32.26 (8.87)	0.265	32.72 (8.56)	32.33 (8.79)	32.22 (6.08)	0.972
LDL-C, (mg/dl)	109.66 (26.43)	127.14 (35.24)	123.07 (38.40)	0.088	117.38 (35.47)	119.20 (33.80)	125.21 (31.84)	0.732
HDL-C, (mg/dl)	48.58 (8.27)	46.23 (10.33)	47.52 (9.44)	0.590	48.68 (9.90)	47.29 (9.49)	45.50 (8.14)	0.520
Cholesterol, (mg/dl)	179.27 (30.28)	194.51 (37.54)	191.87 (40.42)	0.191	186.39 (37.50)	188.33 (37.10)	191.61 (33.10)	0.890
TG, (mg/dl)	105.18 (37.71)	105.74 (42.17)	106.39 (45.88)	0.994	101.65 (49.15)	109.21 (33.22)	104.50 (44.42)	0.737
AIP	−0.04 (0.18)	−0.02 (0.23)	−0.04 (0.26)	0.890	−0.08 (0.26)	−0.01 (0.18)	−0.03 (0.24)	0.399
Glucose, (mg/dl)	91.76 (9.23)	92.94 (13.38)	89.13 (9.55)	0.440	92.32 (10.90)	91.11 (11.61)	91.22 (10.60)	0.893
Insulin, U/mL	14.50 (9.45, 26.85)	14.50 (9.10, 25.50)	16.20 (8.90, 24.20)	0.937	15.40 (8.70, 22.70)	14.80 (9.65, 25.95)	14.25 (5.95, 25.90)	0.898
HOMA-IR	3.26 (1.99, 6.24)	3.91 (1.95, 5.70)	3.44 (1.96, 5.44)	0.874	3.68 (1.95, 5.32)	3.34 (2.09, 5.62)	4.12 (1.38, 6.28)	0.943
QUICKI	0.33 (0.03)	0.32 (0.03)	0.33 (0.03)	0.724	0.33 (0.03)	0.32 (0.03)	0.33 (0.04)	0.561
SBP(mmHg)	112.85 (17.49)	114.31 (14.82)	117.09 (13.79)	0.596	114.06 (15.88)	113.79 (14.99)	116.83 (16.78)	0.750
DBP(mmHg)	75.45 (13.90)	77.09 (9.31)	78.57 (12.40)	0.629	75.77 (11.40)	75.69 (10.78)	81.50 (14.36)	0.170
MetS (%)	28.0	40.0	32.0	0.260	44.0	32.0	24.0	0.643
FADS2 (%)				0.537				0.897
CC	33.3	50.0	16.7		37.5	37.5	25.0	
CT	42.9	26.2	31.0		33.3	45.2	21.4	
TT	16.7	50.0	33.3		33.3	33.3	33.3	

*Data are presented as mean (SD) or median (25 and 75 percentiles)*.

* *Analysis of variance for continuous variables and χ^2^ test for categorical variables. WC, waist circumference; SES, socio-economic status; HOMA-IR, homeostasis model assessment of insulin resistance; LDL-C, low density lipoprotein cholesterol; HDL, high-density lipoprotein-cholesterol; SBP, systolic blood pressure; DBP, diastolic blood pressure; TG, triglyceride; QUICKI, quantitative insulin sensitivity check index; AIP, athrogenic index of plasma; MetS, metabolic syndrome; FM, fat mass; FFM, fat free mass; FADS, fatty acid desaturase*.

A sex-stratified analysis for the association between dietary quality indices (DASH and MDS scores) and FADS2 rs174583 genotypes is shown in Table 5, and as summarized in this table, no significant association was revealed between dietary quality indices and the rs174583 variant in either crude or multivariate-adjusted models, among men and women. Crude and multivariable-adjusted odds ratios and 95% confidence intervals (95% CI) for MetS and cardio-metabolic factors (high cholesterol, high LDL-C, low HDL-C, high TG, elevated blood pressure, hyperglycemia, high HOMA-IR, low QUIKI) across FADS2 rs174583 genotypes are presented in Table 6. There was a significant positive association between the TT genotype and the odds of having high TG when compared to the CC genotype, either in the crude (OR, 3.21; 95% CI, 1.02–10.14) or in the adjusted model 1 (OR, 3.32; 95% CI, 1.00–11.01) and model 2 (OR, 3.58; 95% CI, 1.07–11.97), taking into account various potential confounders.

**Table 5:** Odds ratio (OR) and confidence interval (CI) for the association between dietary quality indices and FADS2 rs174583 genotypes.

	**Men**			**Women**		
	**CC**	**CT**	**TT**	**CC**	**CT**	**TT**
**DASH score (total score)**						
Crude	1(Ref.)	0.93 (0.83–1.04)	1.05 (0.88–1.25)	1(Ref.)	0.99 (0.90–1.12)	1.12 (0.91–1.37)
Model 1^*^	1(Ref.)	0.94 (0.84–1.06)	1.03 (0.86–1.23)	1(Ref.)	1.00 (0.89–1.12)	1.12 (0.90–1.38)
Model 2^**^	1(Ref.)	0.94 (0.84–1.06)	1.03 (0.86–1.23)	1(Ref.)	1.00 (0.89–1.12)	1.12 (0.90–1.38)
**MDS (total score)**						
Crude	1(Ref.)	0.72 (0.49–1.06)	1.09 (0.61–1.93)	1(Ref.)	0.93 (0.69–1.26)	1.06 (0.62–1.83)
Model 1	1(Ref.)	0.75 (0.50–1.11)	1.06 (0.59–1.91)	1(Ref.)	0.93 (0.68–1.26)	0.97 (0.56–1.67)
Model 2	1(Ref.)	0.75 (0.50–1.11)	1.06 (0.59–1.92)	1(Ref.)	1.00 (0.72–1.40)	1.00 (0.57–1.76)

*The multivariate multinomial logistic regression was used for estimation of ORs and confidence interval (CI)*.

* *Adjusted for age, physical activity and socio-economic status*.

** *Additionally adjusted for waist circumference*.

**Table 6:** Odds ratio (OR) and confidence interval (CI) for FADS2 rs174583 genotypes and cardio-metabolic risk factors^a^ of participants.

	**FADS2**		
	**CC**	**CT**	**TT**
**MetS**^**b**^			
Crude	1 (Ref.)	0.68 (0.33–1.39)	1.81 (0.59–5.52)
Model 1	1 (Ref.)	0.76 (0.36–1.61)	1.64 (0.52–5.23)
Model 2	1 (Ref.)	0.88 (0.40–1.93)	1.74 (0.53–5.70)
**High Cholesterol**			
Crude	1 (Ref.)	1.50 (0.72–3.12)	2.69 (0.86–8.37)
Model 1	1 (Ref.)	1.52 (0.72–3.23)	2.61 (0.82–8.32)
Model 2	1 (Ref.)	1.41 (0.65–3.02)	2.55 (0.79–8.17)
**High LDL-C**			
Crude	1 (Ref.)	1.06 (0.05–2.27)	0.82 (0.25–2.73)
Model 1	1 (Ref.)	1.19 (0.54–2.61)	0.73 (0.22–2.51)
Model 2	1 (Ref.)	1.13 (0.51–2.53)	0.68 (0.20–2.36)
**Low HDL-C**			
Crude	1 (Ref.)	1.03 (0.53–2.01)	3.32 (0.96–11.50)
Model 1	1 (Ref.)	1.00 (0.51–1.99)	3.24 (0.92–11.38)
Model 2	1 (Ref.)	1.07 (0.53–2.15)	3.37 (0.95–12.00)
**High TG**			
Crude	1 (Ref.)	0.61 (0.26–1.43)	**3.21 (1.02–10.14)**^**c**^
Model 1	1 (Ref.)	0.69 (0.29–1.66)	**3.32 (1.00–11.01)**^**c**^
Model 2	1 (Ref.)	0.77 (0.32–1.87)	**3.58 (1.07–11.97)**^**c**^
**High blood pressure**			
Crude	1 (Ref.)	0.73 (0.35–1.51)	0.65 (0.19–2.28)
Model 1	1 (Ref.)	0.85 (0.40–1.82)	0.58 (0.16–2.10)
Model 2	1 (Ref.)	0.98 (0.44–2.19)	0.53 (0.14–2.08)
**Hyperglycemia**			
Crude	1 (Ref.)	1.21 (0.50–2.89)	2.94 (0.87–9.95)
Model 1	1 (Ref.)	1.32 (0.54–3.22)	2.72 (0.78–9.41)
Model 2	1 (Ref.)	1.37 (0.55–3.44)	2.67 (0.75–9.55)
**High HOMA-IR**			
Crude	1 (Ref.)	1.05 (0.53–2.08)	0.88 (0.29–2.69)
Model 1	1 (Ref.)	1.06 (0.53–2.12)	0.83 (0.27–2.58)
Model 2	1 (Ref.)	1.10 (0.54–2.24)	0.81 (0.26–2.54)
**Low QUIKI**			
Crude	1 (Ref.)	1.42 (0.43–4.63)	0.79 (0.14–4.36)
Model 1	1 (Ref.)	1.53 (0.46–5.12)	0.81 (0.15–4.55)
Model 2	1 (Ref.)	1.69 (0.50–5.75)	0.80 (0.14–4.53)

*The multivariate multinomial logistic regression across Tertiles of dietary DASH score and MDS was used for estimation of ORs and confidence interval (CI). Model1, Adjusted for age, sex and physical activity. Model 2, Further adjusted for waist circumference and socio-economic status*.

a *Defined as having high cholesterol (total cholesterol ≥ 220 mg/dl), high LDL-C (≥160 mg/dl), low HDL-C (<40 mg/dl for men and <50 mg/dl for women), high TG (≥ 150 mg/dl), high blood pressure (systolic/diastolic blood pressure ≥130/85 mmHg), hyperglycemia (fasting blood sugar ≥100 mg/dl), high HOMA-IR (> 2.6) and low QUIKI (<38)*.

b *Defined as the presence of 3 of the following components: (1) abdominal adiposity (waist circumference > 88 cm); (2) low serum HDL cholesterol (<50 mg/dL); (57) high serum triacylglycerol (≥150 mg/dL); (3) elevated blood pressure (≥130/85 mm Hg); (4) abnormal glucose homeostasis (fasting plasma glucose ≥110 mg/dL). HOMA-IR, homeostasis model assessment of insulin resistance; LDL-C, low density lipoprotein cholesterol; HDL-C, high-density lipoprotein-cholesterol; TG, triglyceride; QUICKI, quantitative insulin sensitivity check index; MetS, metabolic syndrome*.

c *Indicates statistically significant values as P < 0.05*.

**Figure 2:**
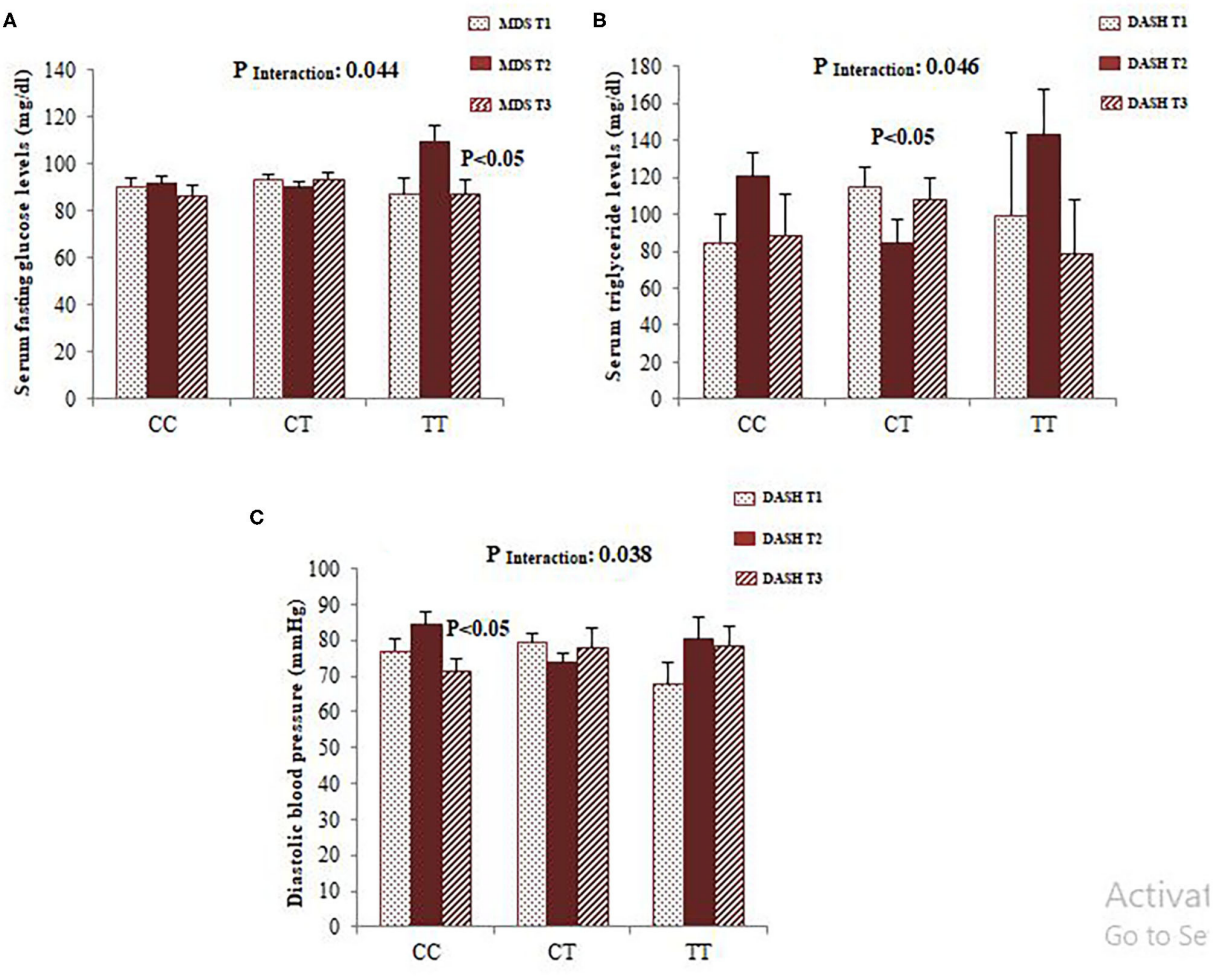
Interaction between FADS2 rs174583 and MDS on the serum concentration of glucose **(A)** and interaction between FADS2 rs174583 and DASH score on the serum concentration of triglyceride **(B)** among women. Interaction between FADS2 rs174583 and DASH score on DBP among men **(C)**. The bars indicate mean. Error bars: SE of means. *P*-values of interactions were adjusted for age, WC, physical activity, and socio-economic status.

To confirm whether FADS2 rs174583 polymorphism interacts with dietary quality indices to modulate cardio-metabolic risk factors, we carried out covariance analyses and illustrated the significant interactions, as shown in Figure 2. Among the female participants, a significant interaction was observed between rs174583 and adherence to the DASH score in relation to serum TG concentration, and this result remained significant even after controlling for confounding variables (*P*_Interaction_ = 0.046). Female CT-genotype carriers who were assigned to the second tertile of DASH compared with those in the first tertile had the lower TG level (*P* < 0.05) (Figure 2). Accordingly, in the female group, another significant interaction was revealed between adherence to the MDS score and rs174583 polymorphism on serum glucose levels (*P*_Interaction_ = 0.044), such that the lowest mean of glucose level was observed in homozygous minor subjects (TT) in the third tertile of MDS, in comparison with other tertiles of this dietary index (*P* < 0.05). Among the male group, no FADS–diet interaction was found except for DASH and rs174583 in relation to diastolic blood pressure (DBP) (*P* interaction = 0.038), where homozygous carriers of the major allele who were assigned to the highest tertile of DASH had significantly lower DBP levels than those in the second tertile (*P* < 0.05).

## Discussion

The current study, based on our knowledge, is the first attempt to study the interactions between FADS2 gene polymorphism (rs174583) and dietary quality indicators in relation to cardio-metabolic risk factors. We found consistently significant interactions between adherence to DASH and rs174583 polymorphism of the FADS2 gene in relation to serum TG level among female subjects; the CT heterozygote which was assigned to the second tertile of DASH had the lower TG level than those who were in the first tertile. Additionally, adherence to the MDS modified the effects of FADS2 rs174583 polymorphisms on levels of glucose in women; minor homozygote carriers with the highest adherence to MDS had the lowest glucose concentrations. On the other hand, being in the highest tertiles of DASH could indicate favorable effects in decreasing DBP in male homozygous carriers of the major allele. Another main finding in this study was that FADS2 rs174583 was associated with higher odds of having high serum TG in multivariate-adjusted models. The minor allele frequency of FADS2 rs174583 in our research (36 %) was lower than reported in a sample of the Taiwanese (58) and European (HapMap database) population. The reason for the differences in allele frequencies by population or ethnic group is not fully understood but could be, in part, explained by differences in sample size, study design, lifestyle, and other demographic characteristics among studies.

In the present study, we revealed novel evidence from an aspect of gene–diet interaction that the associations between the FADS2 rs174583 polymorphism and cardio-metabolic risk factors depend on the diet consumed. Therefore, it seems that improving adherence to dietary quality indices can attenuate the genetic association with metabolic risk factors. As elucidated above, a good adherence to both DASH and MDS could show favorable effects in reducing some of the metabolic risk factors including TG and glucose in female minor allele carriers and DBP in male major allele carriers. Mechanisms involved in these gender-dependent differences are not clear; however, these discrepancies can be attributed to heterogeneities in regional depots of adipose tissue and hormonal status (59). Although there is no relevant published study regarding the FADS2 rs174583–diet interaction to compare our results with, a cross-sectional genome-wide association study (GWAS) in the Korean population reported significant interactions between total fat intake and the FADS1 and haplotype of FADS1 and FADS2 in relation to the MetS risk; carriers of the FADS1 rs174547 and FADS2 rs2845573 minor alleles have a greater predisposition to MetS and its components such as dyslipidemia, and, on the other hand, intermediate intake of dietary fat protected carriers of the FADS1 major alleles against the risk of MetS (60). Another investigation in Swedish adults indicated that minor allele carriers of FADS1 had lower LDL-cholesterol concentrations, following the consumption of a low n-3 long-chain polyunsaturated fatty acid diet (61). Evidence emerging from human-based studies has reported that genetic variation in FADS genes through changes in enzyme activity, which is usually estimated using product-to-substrate ratios, and plasma and erythrocyte fatty acid composition affect disease risk factors (62). Accordingly, several previous studies have reported a lower risk of mortality, insulin resistance, and obesity with reduction of the D6D activity (63, 64). Likewise, the associations have been observed between polymorphisms in FADS genes and high D6D activity (65), as well as between these variants and increased levels of inflammatory markers and fasting insulin and a higher risk of coronary artery disease (66). Thus, our results regarding the association between rs174583 polymorphism and greater odds of having TG concentrations are consistent with the results of other previous studies, where the FADS2 rs174583 polymorphism was associated with an increased risk of overweight and its related consequences (17). The biological mechanisms underlying how FADS genetic variations contribute to disease development have not yet been elucidated. However, it has hypothesized that these variants may impair desaturase activity and lead to a decrease in n-3 LC-PUFA in the red blood cell (RBC) and consequently deleterious metabolic effects (58).

Altogether, our results suggest that adherence to the healthy dietary pattern could modify the genetic association with some of the cardio-metabolic parameters. Substantial evidence supports a protective effect of improved adherence to the diet quality scores, which represent a broader picture of dietary intake, on health outcomes (26). For instance, Saraf-Bank et al. in a sample of Iranian subjects revealed that higher healthy eating index (HEI) 2010 scores were inversely associated with lower risk of MetS and its components (67). Likewise, similar results were observed in our previous study in which high adherence to HEI was found to be inversely associated with MetS risk (44). Therefore, it is not surprising that high compliance with these healthy dietary patterns suppresses the adverse influence of greater genetic susceptibility to cardio-metabolic risk factors in carriers of the FADS2 risk allele; in other words, people appear to be more prone to the beneficial effects of improving diet quality. The precise underlying mechanisms behind these interactions are not fully understood. Nevertheless, the favorable modulating effects of these indices could be explained by their components which are accompanied with high intake of fruits, vegetables, whole grains, seafood and plant proteins, nuts, and fiber and low intake of saturated fatty acids (68). In this regard, extensive evidence from epidemiologic studies has revealed the inverse associations of fruit, vegetables, and unsaturated fats with nutrition-related health conditions (69). In the present study, we found that greater adherence to the DASH score was associated with lower AIP and TG levels in men. These findings were in concordance with previous studies that reported a protective role for DASH diet against cardio-metabolic risk factors. A number of meta-analyses of RCT have revealed inverse relationships between DASH and CVD risk factors (70, 71). Additionally, Maddock et al. confirmed that long-term adherence to a DASH-type diet is related to a favorable cardiovascular-risk profile in adulthood (72). As mentioned above, since this diet is characterized by a higher consumption of healthy food items such as whole grain, fruits and vegetables, low-fat dairy products, lean meat, poultry and fish, nuts, and legumes which are rich dietary sources of potassium, magnesium, calcium, and dietary fiber, health benefits of excellent adherence with the DASH score are of no surprise (73).

A major strength of this study is that we, for the first time, found significant interactions between healthy dietary patterns and genetic predisposition in relation to cardio-metabolic risk factors, which is highly novel; therefore, our findings emphasize the importance of considering the influence of gene–diet interactions in relation to health outcomes. Thoroughly, the identification and better understanding of gene–diet interactions may open new opportunities for developing precision nutrition strategies based on subjects' genotype for the prevention and control of diseases. Moreover, diet quality scores provide comprehensive measures of diet than the single-component approach. Another strength of the present study was that we applied a reliable and validated FFQ for dietary assessment. Nonetheless, some limitations of this investigation need to be highlighted. First, although the cross-sectional design of the study makes causal inferences impossible, this research helps to generate the hypothesis to be studied more rigorously using cohort studies or other types of study. Second, under-reporting of dietary intake which is significant among obese people may cause misclassifications in dietary variables and ultimately this phenomenon can result in bias in the diet–disease relationship (74). Nevertheless, we removed the lower and higher extreme values of dietary intake. Third, despite that we have carefully adjusted for multiple confounders in the analyses, residual confounding by other unmeasured or unknown variables could not be fully eliminated. Fourth, it might be limited by the relatively small sample size. Thus, our finding should be interpreted with caution and replication in large epidemiological cohort studies is needed. Fifth, we have to mention that only one variant from a single gene was analyzed in this study while a number of other candidate genes have been implicated in the pathogenesis of the obesity phenotype and its dependent complications. Last, our conclusions may not be generalized to the general population as the present study was performed in Tabriz with different cultures, dietary intakes, and other lifestyle factors.

## Conclusion

In conclusion, we found for the first time the statistically significant interactions of FADS2 rs174583 with adherence to the dietary quality indicators (DASH and MDS) in relation to cardio-metabolic risk factors in both women and men. Female carriers of the risk allele with higher adherence to DASH and MDS had lower adjusted means of TG and glucose concentrations, respectively. Likewise, a similar interaction was observed for DBP in male homozygous carriers of the major allele. Additionally FADS2 rs174583 polymorphism was associated with higher odds of having high serum TG. Therefore, our findings highlight the importance of improving adherence to a healthy diet in prevention of obesity-dependent complications especially in subjects with greater genetic susceptibility. Subsequently, prospective cohort studies are needed to confirm the results of our study.

## Data Availability Statement

The datasets generated for this study are available on request to the corresponding author.

## Ethics Statement

The studies involving human participants were reviewed and approved by IR.TBZMED.REC.1399.062. The patients/participants provided their written informed consent to participate in this study.

## Author Contributions

MK contributed to the data collection, manuscript writing, performed the statistical analysis, and data interpretation. MA conceptualized and designed the study. Moreover, MA revised the manuscript and approved the final manuscript as submitted. LN was involved in lab works. All authors contributed to the article and approved the submitted version.

## Conflict of Interest

The authors declare that the research was conducted in the absence of any commercial or financial relationships that could be construed as a potential conflict of interest.
